# Variation in attitudes toward diagnosis and medication of ADHD: a survey among clinicians in the Norwegian child and adolescent mental health services

**DOI:** 10.1007/s00787-022-02110-7

**Published:** 2022-11-19

**Authors:** Ingvild Lyhmann, Tarjei Widding-Havneraas, Henrik Daae Zachrisson, Ingvar Bjelland, Ashmita Chaulagain, Arnstein Mykletun, Anne Halmøy

**Affiliations:** 1 Centre for Research and Education in Forensic Psychiatry, Haukeland University Hospital, Bergen, Norway; 2 Department of Clinical Medicine, University of Bergen, Bergen, Norway; 3 Department of Special Needs Education, University of Oslo, Oslo, Norway; 4 Division of Psychiatry, Haukeland University Hospital, Bergen, Norway; 5 UiT - The Arctic University of Norway, Tromsø, Norway; 6 Nordland Hospital Trust, Centre for Work and Mental Health, Bodø, Norway; 7 Division of Mental Health, Norwegian Institute of Public Health, Oslo, Norway

**Keywords:** ADHD, Survey, Clinician attitude, Practice variation, Diagnosis, Medication

## Abstract

Prevalence and medication rates of ADHD vary geographically, both between and within countries. No absolute cutoff exists between ADHD and normal behavior, making clinician attitudes (leading to local practice cultures) a potential explanation for the observed variation in diagnosis and medication rates. The objective of this study was to describe variation in attitudes toward diagnosis and medication of ADHD among clinicians working in child and adolescent mental health services (CAMHS). We hypothesized that attitudes would vary along a spectrum from “restrictive” to “liberal”. We also explored whether differences in attitudes between clinicians were related to professional background and workplace (clinic). A survey in the form of a web-based questionnaire was developed. All CAMHS outpatient clinics in Norway were invited. Potential respondents were all clinicians involved in diagnosing and treating children and adolescents with ADHD. To investigate the existence of attitudes toward diagnosis and medication as latent constructs, we applied confirmatory factor analysis (CFA). We further examined how much of variance in attitudes could be ascribed to profession and clinics by estimating intraclass correlation coefficients. In total, 674 respondents representing 77 (88%) of the clinics participated. We confirmed variation in attitudes with average responses leaning toward the “restrictive” end of the spectrum. CFA supported “attitude toward diagnosis” and “attitude toward medication” as separate, and moderately correlated (*r* = 0.4) latent variables, representing a scale from restrictive to liberal. Professional background and workplace explained only a small part of variance in these attitudes.

**Supplementary Information:**

The online version contains supplementary material available at 10.1007/s00787-022-02110-7.

## Introduction

Attention-deficit/hyperactivity disorder (ADHD) is characterized by symptoms of inattention and/or hyperactivity and impulsivity that are inappropriate for the individual’s age and developmental level [2]. With an estimated worldwide prevalence of 5.9% [3], it is one of the most commonly diagnosed mental disorders in children.

Reported rates of ADHD vary considerably between studies from different countries [3], and even regionally, within countries [4–7]. In the case of regional differences, the observed variation cannot be caused by study methodology as comparisons are based on official numbers of registered diagnoses. In Norway, a recent study has empirically demonstrated that the large geographic variation in diagnosis rates in the country cannot be explained by a correspondingly varying symptom load in the population [8]. Likewise, despite close similarities between ADHD treatment guidelines internationally, medication rates also differ markedly both between and within countries [5–7, 9–11].

ADHD symptoms exist on a continuum, with no absolute cutoff between clinical diagnosis and “normal” functioning [12, 13]. Moreover, a wide range of conditions can have manifestations resembling ADHD symptoms [14–16]. As for other mental disorders, there are no biomarkers or decisive tests available for objectively confirming an ADHD diagnosis in clinical practice. Observed symptoms must thus be assessed by a clinician in terms of their onset, course, severity, and resulting functional impairment to decide whether diagnostic criteria are fulfilled and if an underlying vulnerability is the most likely cause. In cases bordering on the threshold for ADHD diagnosis, the diagnostic decision will to some extent be a result of the individual clinician’s judgment.

Unnecessarily receiving a diagnosis and medication or being deprived of an appropriate diagnosis and possibly beneficial treatment both have potential detrimental effects in a life course perspective [17–20]. The long-lasting debate in the research literature and among practicing clinicians demonstrates concerns over both alternatives, with experts emphasizing different aspects of this dilemma [13]. Central points of concern regarding excessive diagnosis include the various effects of stigma and labeling [17, 18], as well as the general societal medicalization of childhood. Apprehensions associated with overtreatment span from worries about the immediate side effects of medications to the current lack of knowledge about the long-term consequences of their use [20]. On the other hand, receiving a formal diagnosis and treatment may reduce negative consequences of ADHD symptoms [19], as it offers a framework for increased understanding of the individual’s challenges and provides access to medication as a means of potential symptom relief [17]. How clinicians balance these considerations will presumably influence their clinical decisions, which may range from taking a restrictive stance aiming to minimize the use of diagnoses and/or medication, to being more liberal in terms of diagnosing or recommending medication even in less clear-cut cases.

Variation in medical practice, both at the individual and group level (i.e., local practice cultures), is a well-documented phenomenon [21]. Some previous research has examined different aspects of clinicians’ attitudes toward ADHD and how this may influence clinical practice [see e.g., 22–27], though mostly targeting physicians, often in the role of gatekeepers to specialized mental health services. In Norway, ADHD diagnosis and treatment are decided by clinicians working in a specialized and interdisciplinary healthcare setting, where the therapist in charge will often be a clinical psychologist.

The objective of the current study was to describe variation in attitudes toward diagnosis and medication of ADHD among clinicians working in the Norwegian child and adolescent mental health services (CAMHS) outpatient clinics. Further, we wanted to explore if differences in attitudes between clinicians were related to professional background or workplace (clinic). We hypothesized that attitudes vary on a spectrum from “restrictive” to “liberal”. We proposed that such differences might influence the management of children on the margin of ADHD diagnosis, and might help explain the geographical variance in rates of diagnosis and medication of ADHD.

## Methods

### Survey development

To investigate our objective, we developed a survey aimed at measuring clinicians’ attitudes toward diagnosis and medication of ADHD as latent constructs. A team consisting of two clinical psychologists (AM, IL) and two psychiatrists with extensive clinical experience with ADHD patients (IB, AH) designed the questionnaire. To further improve validity and reliability of the items, five external clinicians with special interest in ADHD tested the survey during its development and were interviewed to collect feedback on points of improvement. In addition, the survey was piloted in one clinic.

### Survey design

The survey was developed as a one-time, self-administered, web-based questionnaire. Questions were closed-ended with four or six Likert-scaled, forced choice options (i.e., no neutral response possible). Items were developed assuming variation from restrictive to liberal attitudes toward ADHD diagnosis and medication.

Comment boxes for optional supplementary comments were provided for each item. All except for two background questions were possible to skip. To maximize response rate, we aimed for a survey that was quick to complete (< 10 min). The survey also included some items as part of a broader investigation of ADHD practice in the clinics that are not relevant to the objective in this article and thus not presented here. The complete, translated survey can be found in Appendix A.

To explore variation in clinician attitudes, eight items were used in analyses. To ease communication of results, all items have been given labels that are indicated in italics throughout the text. Three survey items covered background information about respondents (*profession*/educational background, work *experience*, and *frequency* of contact with ADHD patients, Table 1). Three items involved attitudes regarding diagnosis of ADHD (*certainty* when diagnosing, hypothetical prevalence in an *ideal* world, Table 2; *await* making a diagnostic decision, Table 3). One item considered treatment of ADHD (statements about *medication*, Table 4), and one item was originally intended to cover both diagnosis and medication (*over/undertreatment* in Norway today, Table 2). Two of these items (*await*, *medication*) consisted of a question followed by several statements (“sub-items”) to which respondents indicated to which extent they agreed. See Tables 1, 2, 3 and 4 for wording and options.

**Table 1:** Sample characteristics

*Profession*^a^	%	*n* = 674
Profession/educational background		
Medical doctor/child and adolescent psychiatrist in training	6.2	42
Child and adolescent psychiatrist	12.6	85
Clinical psychologist	27.2	183
Clinical psychologist with specialization in child and adolescent psychology	25.8	174
Nurse	1.8	12
Learning disability nurse	1.2	8
Social worker	6.1	41
Educationalist	14.4	97
Other	4.7	32

*Experience*	%	*n* = 672
Total work experience in child and adolescent mental health services		
< 5 years	40.0	269
5–15 years	36.9	248
> 15 years	23.1	155

*Frequency*^a^	%	*n* = 674
How often do you see patients with suspected or diagnosed ADHD?		
Approx. daily	30.0	202
Approx. weekly	54.0	364
Approx. monthly	12.8	86
Less [than monthly]	3.3	22

^a^Required question

**Table 2:** Items covering attitudes concerning thresholds for diagnosis and treatment

*Certainty*	%	95 % CI	*n* = 661
Opinions differ regarding how certain one needs to be in order to diagnose ADHD. What is your opinion?			
It must be considered >95 % likely that the patient has ADHD^−^	45.8	42.0–49.6	303
It must be considered >75 % likely that the patient has ADHD	50.4	46.6–54.2	333
It must be considered >50 % likely that the patient has ADHD	1.8	0.8–2.8	12
Diagnosis can be made on an even more uncertain basis if it is assumed that the patient will benefit from pharmacological treatment^+^	2.0	0.9–3.0	13

*Over/undertreatment*	%	95 % CI	*n* = 657
There is disagreement regarding the best way to manage children with ADHD symptoms in the child and adolescent mental health services. Some fear that we are medicalizing normal conditions and that too many receive a diagnosis and medication. Others think the opposite, suggesting more children should be prescribed medication What is your opinion about the situation in Norway today?			
Overtreatment is most prevalent^−^	26.8	23.4–30.2	176
Undertreatment is most prevalent^+^	4.7	3.1–6.3	31
Both over and undertreatment occurs, about equally frequently	60.3	56.5–64.0	396
Neither over nor undertreatment occurs to a significant degree	8.2	6.1–10.3	54

*Ideal*	%	95 % CI	*n* = 673
Imagine the ideal scenario where all children live under optimal psychosocial conditions, having involved and caring caregivers, receiving appropriate support and accommodations in school, etc. Assume also that health and social services has access to ample resources and competent professionals. Compared to today, what do you think the prevalence of ADHD among children and adolescents would be in the ideal scenario?			
Considerably higher^+^	0.1	0.0–0.4	1
Somewhat higher	2.2	1.1–3.3	15
Unchanged	4.9	3.3–6.5	33
Somewhat lower	53.3	49.6–57.1	359
Considerably lower	39.1	35.4–42.8	263
ADHD would not exist^−^	0.3	0.0–0.7	2

Symbols designate the option scored as most restrictive (^−^) and most liberal (^+^)

**Table 3:** Item with sub-items covering attitude toward diagnosis (DA)

*Await* Sometimes the clinical picture corresponds to ADHD but ruling out alternative causes for the symptoms is difficult. In the following cases, how often will you postpone making the diagnostic decision?						
		Always^−^	Often	Sometimes	Never^+^	Total (*n*)
(D1)	Considerable psychosocial challenges	163	398	108	2	671
		24.3 %	59.3 %	16.1 %	0.3 %	
		(21.0–27.5)^b^	(55.6–63.0)	(13.3–18.9)	(0.1–0.7)	
(D2)	History or presence of trauma	216	366	86	2	670
		32.2	54.6	12.8	0.3	
		(28.7–35.8)	(50.9–58.4)	(10.3–15.4)	(0.0–0.7)	
(D3)	Considerable health problems in close family	48	244	348	26	666
		7.2	36.6	52.3	3.9	
		(5.2–9.2)	(33.0–40.3)	(48.5–56.0)	(2.4–5.4)	
(D4)	Diagnosed sensory deficit (sight, hearing)	75	266	285	24	650
		11.5	40.9	43.8	3.7	
		(9.1–14.0)	(37.1–44.7)	(40.0–47.7)	(2.2–5.1)	
(D5)	Diagnosed neurological conditions	108	319	228	6	661
		16.3	48.3	34.5	0.9	
		(13.5–19.2)	(44.5–52.1)	(30.9–38.1)	(0.2–1.6)	
(D6)	Intellectual functioning deviating from normal range	122	353	189	5	669
		18.2	52.8	28.3	0.7	
		(15.3–21.2)	(49.0–56.5)	(24.8–31.7)	(0.1–1.4)	
(D7)	Other diagnosed developmental disorders	81	301	278	5	665
		12.2	45.3	41.8	0.8	
		(9.7–14.7)	(41.5–49.0)	(38.1–45.6)	(0.1–1.4)	
(D8)	Other (please specify)	27	33	55	102	217
		12.4	15.2	25.3	47.0	
		(8.1–16.8)	(10.4–20.0)	(19.6–31.1)	(40.4–53.6)	

^b^95% CI Alpha: 0.86

Symbols designate the option scored as most restrictive (^−^) and most liberal (^+^)

**Table 4:** Item with sub-items covering attitude toward medication (MA)

*Medication* We would like your opinions on the following statements regarding treatment of ADHD						
		Strongly agree	Somewhat agree	Somewhat disagree	Strongly disagree	Total (*n*)
(M1)	Medication is the only real option in the treatment of ADHD^+^	3	65	178	423	669
		0.4 %	9.7 %	26.6 %	63.2 %	
		(0.0–1.0)^c^	(7.5–12.0)	(23.3–30.0)	(59.6-66.9)	
(M2)	If the patient responds well to medication, there is no need to initiate additional interventions^+^	4	16	108	545	673
		0.6	2.4	16.0	81.0	
		(0.0–1.2)	(1.2–3.5)	(13.3–18.8)	(78.0–83.9)	
(M3)	I am worried about the long-term consequences of using medication in ADHD treatment^−^	69	257	240	101	667
		10.3	38.5	36.0	15.1	
		(8.0–12.7)	(34.8–42.2)	(32.3–39.6)	(12.4–17.9)	
(M4)	Medication is a prerequisite for enabling psychosocial interventions to work^+^	12	193	308	156	669
		1.8	28.8	46.0	23.3	
		(0.8–2.8)	(25.4–32.3)	(42.3–49.8)	(20.1–26.5)	
(M5)	The side effects of ADHD medications are stronger than many clinicians acknowledge^−^	24	230	316	75	645
		3.7	35.7	49.0	11.6	
		(2.3–5.2)	(32.0–39.4)	(45.1–52.9)	(9.2–14.1)	
(M6)	The use of medications in the treatment of children should be reduced as much as possible^−^	124	263	222	58	667
		18.6	39.4	33.3	8.7	
		(15.6–21.5)	(35.7–43.1)	(29.7–36.9)	(6.6–10.8)	
(M7)	Medication appears in many instances to become an excuse for institutions and adults surrounding the child not to take further action, so that other important interventions are neglected^−^	151	379	107	28	665
		22.7	57.0	16.1	4.2	
		(19.5–25.9)	(53.2–60.8)	(13.3–18.9)	(2.7–5.7)	
(M8)	If the patient responds well to psychosocial interventions, initiating medication is unnecessary^−^	299	249	110	12	670
		44.6	37.2	16.4	1.8	
		(40.9–48.4)	(33.5–40.8)	(13.6–19.2)	(0.8–2.8)	
(M9)	Psychosocial interventions are a prerequisite for enabling medication to work^−^	223	290	126	30	669
		33.3	43.3	18.8	4.5	
		(29.8–36.9)	(39.6–47.1)	(15.9–21.8)	(2.9–6.1)	

^c^95% CI Alpha: 0.65

Symbols denote whether “Strongly agree” is scored as most restrictive (^−^) or most liberal (^+^) for each sub-item

### Respondents

We aimed to include the whole population of clinicians, including all healthcare professionals of various educational backgrounds, currently involved in diagnosing and treating ADHD in the Norwegian CAMHS outpatient clinics. This represents most clinicians working with ADHD among children and adolescents in Norway, as the private health sector in child psychiatry is negligible.

### Data collection procedure

As there is no central registry of employees in the 88 CAMHS outpatient clinics in Norway, we approached the heads of all the clinics and asked them to forward the survey to relevant employees in their unit. Each clinic received a unique link, enabling us to give feedback to the clinics on their average scores compared to national data (provided a response rate of > 50%).

The survey was accessible from September to December 2020. A reminder was sent to all heads of clinics two weeks after initial distribution. Clinics that still had not returned any responses a few weeks after the reminder received personalized follow-up emails or a call.

### Statistical modeling and analysis

Answers to survey items concerning attitudes were scored along the hypothesized dimension with low scores representing restrictive and high scores liberal attitudes. To estimate response rate, we used the coverage of clinicians per 1,000 children in some catchment areas (made available to us by coauthor IB), which should be roughly generalizable to other regions.

Structural equation modeling (SEM) was applied to estimate and examine the relationships between our hypothesized attitude variables. A two-factor confirmatory factor analysis (CFA) was used to estimate the latent constructs of clinicians’ attitude toward diagnosis (DA) and attitude toward medication (MA).

Three survey items concerning attitude toward diagnosis were excluded from the final DA model, one due to high amount of missing (*D8*/other; Table 3) and two because of low factor loadings (*certainty*: $\lambda$ = 0.12; *ideal*: $\lambda$= 0.11; Table 2). DA was thus estimated by seven sub-items of the item *await* (*D1–D7;* Table 3). Residuals were allowed to correlate between some indicators measuring highly related concepts: both *D2*/trauma and *D3*/health problems in the family could also be characterized as *D1*/psychosocial challenges; and *D6 *and *D7* both refer to neurodevelopmental conditions (Table 3).

MA was estimated by seven sub-items of the item *medication* (*M1–M9;* Table4). Two sub-items were excluded due to low factor loadings (*M2*: $\lambda$ = 0.18; *M4*: $\lambda$ = 0.24). Residual correlations were allowed between *M3* and *M5* (both relating to concerns over side effects).

The item *over/undertreatment* (Table 2) was not included in the SEM analysis as its wording in terms of both question and options made it unclear where to fit in the model.

Standardized factor scores for DA and MA were extracted from the two-factor CFA model. The extent of variation in attitudes attributable to profession and clinic level was examined by intraclass correlation coefficients (ICC) in variance-components models, based on factor scores extracted from the factor models. Separate partially latent structural regression models were used to examine associations between experience and frequency (as single exogenous indicators) with DA and MA.

Models were estimated using maximum likelihood with missing values (MLMV) (missing 10% in final models). Model fit was assessed by ${\chi }^{2}$ tests, comparative fit index (CFI), and root mean squared error of approximation (RMSEA). An insignificant ${\chi }^{2},$ CFI > 0.90, and RMSEA < 0.08 indicate a good model fit [28]. Focal strains in the solution were assessed with modification indices. As robustness checks, models were additionally estimated using MLMV with standard errors clustered by clinic; ML with ordinal family and logit link function to account for the Likert-scale of indicators; and mean- and variance-adjusted weighted least squares (WLSMV) with categorical indicators and standard errors clustered by clinic.

The online survey was constructed in Corporater Surveyor, an online survey tool pre-approved by the Data Protection Officer of Haukeland University Hospital. Analysis and data visualization was performed in Stata SE 17, except WLSMV estimated in Mplus v8.1.

## Results

### Descriptive results

In total, 674 respondents representing 77 (88%) of the 88 outpatient clinics in the Norwegian CAMHS completed the survey. Among these, 484 (72%) of respondents were physicians (including child and adolescent psychiatrists) or licensed clinical psychologists, the two professional groups that are authorized to diagnose patients in Norway. The majority (60%) indicated that they have worked in the CAMHS for 5 years of more, and 84% estimated that they see ADHD patients at least weekly (see Table 1). We estimated the response rate among clinicians to be approximately 38%. The 12% of non-responding clinics were geographically spread around the country, representing both urban and rural catchment areas.

As shown in Tables 2, 3 and 4, there was large variation in responses to most attitude items, indicating that the survey successfully measured a spectrum of attitudes from restrictive to liberal. Generally, responses leaned toward the restrictive rather than liberal end of the scale on most survey items. For example, we see from item *ideal* (Table 2) that 92% of respondents think that ADHD would be less prevalent had all children lived under ideal conditions when growing up, suggesting that most clinicians think environmental factors should be carefully considered before making a diagnosis.

The provided comment boxes were frequently utilized by respondents, indicating a high degree of engagement. Comments often emphasized the complexity of decision-making in relation to ADHD symptoms, or moderated the forced responses given to the items. Spontaneous feedback from heads of clinics during the distribution of the survey supported the impression that rates of ADHD diagnosis and medication is considered a controversial and important topic.

Tables 1, 2, 3 and 4 show (translated) questions and options as they were presented in the survey, except for headings in italics and symbols to indicate scoring added for clarity. Except for item *D8*, there was less than 5% missing responses to all items.

### Two-factor model of diagnosis and medication attitudes

In the final SEM model, attitude toward diagnosis (DA) was estimated by seven sub-items of the item *await* (*D1–D7*), describing situations/conditions that might make clinicians hold back in the decision-making process of diagnosing patients with ADHD. Model fit statistics for the one-factor model for DA (MLMV) were considered acceptable (${\chi }^{2}$(11) 65.5, *p* < 0.001, CFI 0.97, RMSEA 0.086). Attitude toward medication (MA) was comprised of seven sub-items consisting of statements about the treatment of ADHD (*medication*). Fit statistics indicated good model fit $({\chi }^{2}$(13) 36.0, *p* < 0.001, CFI 0.95, RMSEA 0.051). All factor loadings were significant.

Overall, DA and MA seemed to reflect separate latent constructs, that are moderately correlated (*r* = 0.4). Model fit statistics for the two-factor model were good (${\chi }^{2}$(72) 188.8, *p* < 0.001, CFI 0.96, RMSEA 0.049). Main results were consistent across model specifications.

**Fig. 1:**
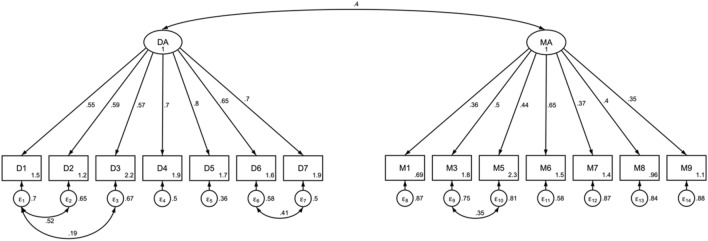
Two-factor SEM model of attitude toward diagnosis (DA; Table 3) and medication (MA; Table 4)

Figure 1 presents the two-factor CFA model with standardized parameters; Tables 3 and 4 show details about the indicators. Standardized and unstandardized parameter estimates can be found in Appendix B.

### Group differences

**Fig. 2:**
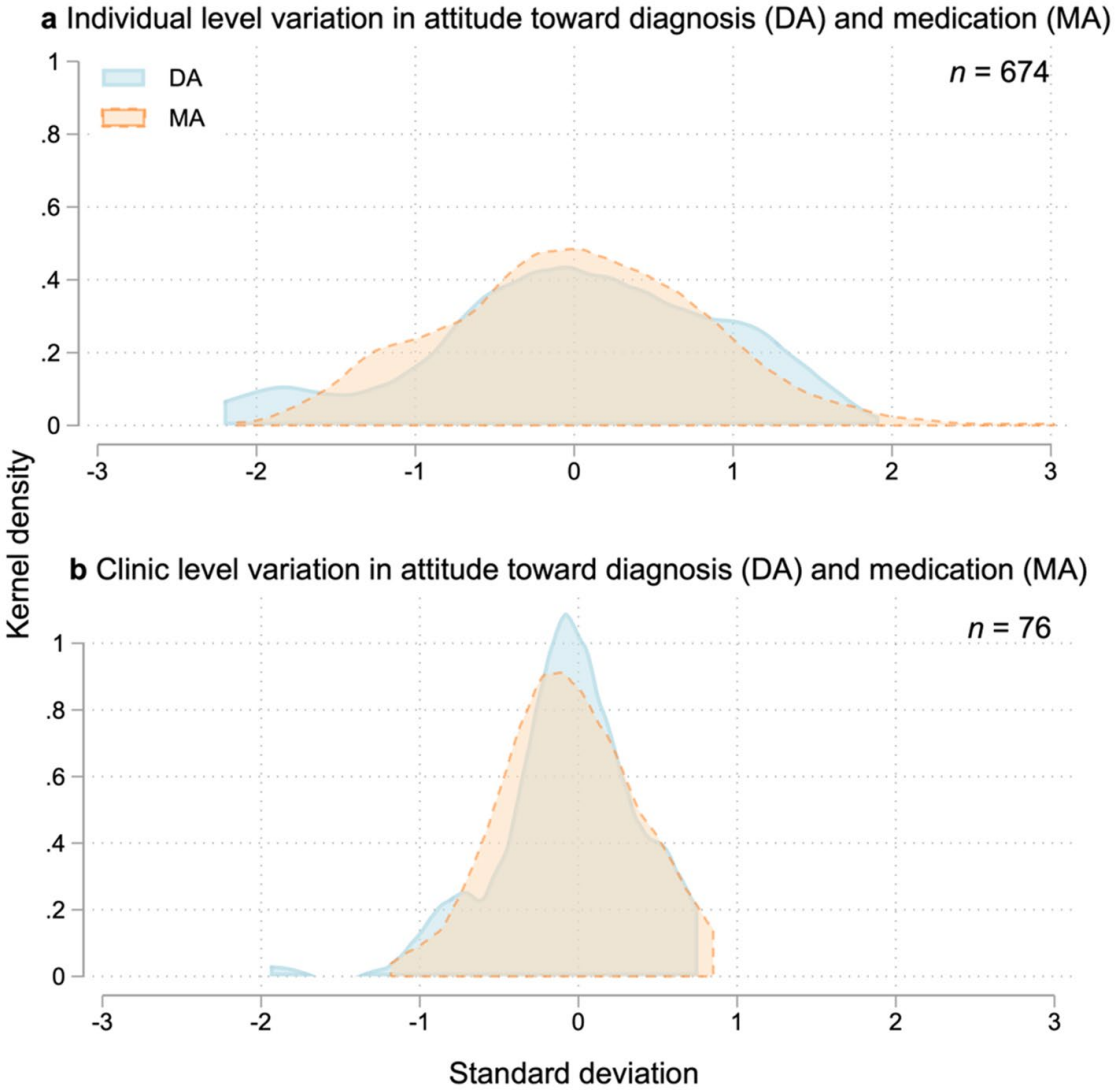
Kernel density plot showing variation in DA and MA scores by clinicians (**a**) and by clinic (**b**)

Intraclass correlations (ICC) indicated that respondents’ professional background constituted 5.2% (95% CI 1.4–17.3) of variation in DA and 1.6% (95% CI 0.2–10.1) in MA. For clinic, ICCs showed that 2.7% (95% CI 0.5–13.7) of variation in DA and 9.8% (95% CI 0.49–18.7) in MA was attributable to the clinic level. Figure 2 shows distribution of the DA and MA factor scores across individuals and clinics, illustrating that individual scores vary more widely than scores based on clinic averages. n = 76 in clinic-level analysis as two closely affiliated clinics were merged to one due to a mix-up of survey links.

Partially latent structural regression models indicated that longer *experience* was associated with somewhat more liberal scores on DA (std. coef. = 0.11, *p* = 0.04), while it did not affect MA scores (std. coef. = 0.03, *p* = 0.66). There was no support for an association between *frequency* and DA (std. coef. = *−*0.06, *p* = 0.18), while lower frequency of contact with ADHD patients led to slightly more restrictive MA scores (std. coef. = *–*0.12, *p* = 0.03).

## Discussion

Our results are based on answers from 674 respondents from the entire population of clinicians working with ADHD in the Norwegian CAMHS outpatient clinics, representing 88% of the clinics in the country. Responses indicated that attitudes toward ADHD diagnosis and medication vary considerably among individual clinicians. Generally, respondents tended to lean toward the restrictive end of the continuum, while a strong liberal attitude was seldom expressed. Only small proportions of variation could be ascribed to professional background or clinic level.

Our CFA supported the hypothesis that attitudes toward diagnosis (DA) and medication (MA) of ADHD can be conceptualized as two separate, but related constructs. The exclusion of the items *certainty* and *ideal* signify that our final DA model became somewhat more narrowly defined than originally planned, consisting only of the sub-items of *await*. Interestingly, when responding to the item *certainty*, many respondents commented that other circumstances must be taken into consideration and mentioned specifically the topics covered in *await,* the subsequent item in the survey. Several respondents commented that they answered on behalf of their team, and that they do not necessarily personally agree with every decision. This could help explain why the items *certainty* and *await* correlated less than expected even if respondents seem to consider them related. The items in the final model were considered an adequate measure of attitude toward diagnosis, indicative of practical diagnostic decision-making. With regard to the MA model, several aspects of attitude toward medication are covered, including beliefs about effect, side effects, and relation to alternative forms of treatment.

Partitioning the total variance into individual and group level revealed that most variance occurred between individuals, and only small proportions (< 10%) of variance could be ascribed to the group level. Clinic affiliation explained considerably more variance in MA than in DA, suggesting that attitude toward medication is more sensitive to the development of local practice cultures than attitude toward diagnosis. In summary, there seems to be large differences in attitudes between individual clinicians, which is likely to be of consequence to their clinical practice. However, as little variance was attributable to the clinic level, the hypothesis that these attitudes influence local practice cultures and cause the geographic variation in ADHD diagnosis and medication rates was not supported by these results.

To our knowledge, this is the first study aiming to estimate and compare clinician attitudes toward ADHD diagnosis and medication by a comprehensive nationwide survey, and that uses statistical analyses that account for potential variation due to clinic affiliation. Our results are in line with the main findings of related previous studies. Using qualitative methods, Kovshoff et al. (2012) found that clinicians describe the process of making decisions in relation to ADHD as challenging, and that they include in their clinical decision a [subjective] consideration of whether the label will be in the best interest of the child [29]. In a follow-up study, the same researchers showed that clinicians differed in their partiality toward medication versus psychosocial intervention, pointing out that this may lead to patients receiving different advice based on provider preference alone [25]. Further, it was observed that clinicians vary in their attitudes regarding the goal of treatment (optimizing versus being satisfied with some “degree of improvement”), which is likely to cause differences in clinical practice. An earlier, related survey on physician perceptions on ADHD medication showed that physicians generally consider ADHD medication to be effective [23]. However, like us, they also found that clinicians were divided in their assessments of the severity and manageability of side effects.

While relevant, the abovementioned studies all include physicians only, albeit of different specialties. To the extent that we can compare our results to studies that include a respondent group including clinicians of diverse educational backgrounds, there seems not to be consistent patterns of group differences in attitudes related to the background variables we have collected. An ambitious international survey [27], although hampered by very low response rate, showed differences between professions in their concerns related to rates of ADHD diagnosis: psychologists were most worried about overdiagnosis, and psychiatrists about underdiagnosis. Dekkers et al. (2021), on the other hand, considered factors related to clinicians’ policy and attitudes toward medication and parent training, and found no difference between clinicians of medical versus non-medical backgrounds in attitudes toward medication or how likely they were to discuss parent training or medication as potential treatment options [26]. This is in line with our finding that professional background explains less than 2% of the observed variation in medication attitude.

### Strengths and limitations

The survey used in this study was designed specifically to extract variation in our hypothesized “restrictive to liberal” attitude dimension. The results, showing large differences between individuals, suggest that we succeeded in this regard. To maximize validity and reliability, the survey was constructed with the involvement of several experienced clinicians, both internal and external to the research group, and was piloted before distribution. The main strength of the study is that we were able to reach out to the entire population of clinicians, and that we achieved a high number of respondents, a satisfactory response rate, and very high geographical, clinic-wise coverage. We have also gathered background variables enabling us to investigate group differences in responses between several relevant groups.

Regarding limitations, we do not know if there are shared characteristics within the group of respondents or non-respondents. It is possible that those heads of clinics and clinicians who chose to invest time in the survey represent those particularly concerned about overdiagnosis and overtreatment of ADHD. Interestingly, despite being of high professional relevance, the topic of this survey seems to be considered sensitive by many. This can potentially have lowered the response rate or skewed answers in a direction considered more socially acceptable by respondents.

Another possible threat to the validity of any survey pertains to the wording and order of questions and options, as this will shape the collected information. As a specific example, the abstract wording of the options of the item certainty seemed to anchor respondents to the first, most restrictive alternatives. If we had reworded to emphasize that 50% certainty corresponds to “finding it more likely that ADHD is the correct diagnosis than not”, the response distribution would likely have been different.

Lastly, we cannot know for certain how much of the actual variation we have succeeded in extracting with our items, and we do not know how respondents would truly act in practical situations. It may be that clinicians who consider themselves similarly restrictive or liberal according to our scale will still make different decisions when faced with comparable cases in clinical practice.

Together, these challenges may explain why our findings did not show clear associations between clinicians’ individual attitudes and group affiliation, leaving the question about clinical practice cultures as a reason for geographical variation in rates of ADHD still unsettled.

## Conclusion

This survey has succeeded in measuring attitudes toward ADHD diagnosis and medication among Norwegian clinicians. We have demonstrated large variation in attitudes between clinicians working in the field, which in turn suggests that clinical practice varies between individual clinicians. However, we did not find large group differences based on clinic affiliation or other background variables. Our results thus do not point to variation in individual attitudes as a main reason for the geographic variation in diagnosis and medication rates. While this alone does not rule out local practice cultures as a reason for the variation, it suggests that any existing practice cultures are driven by forces other than conscious or idealistic differences that we have studied in this survey. As this is an explorative study, its results must be interpreted with some caution. There is need for further research on the potential influence of practice culture on the observed geographic variations in prevalence and treatment of ADHD.

### Supplementary Information

Below is the link to the electronic supplementary material.

Supplementary file1 (PDF 206 KB)

Supplementary file2 (PDF 148 KB)

## Data Availability

Participant consent and relevant approvals/regulations do not allow for data sharing.
