# Supplementary material for: Variation in attitudes toward diagnosis and medication of ADHD: a survey among clinicians in the Norwegian child and adolescent mental health services
Source: Eur Child Adolesc Psychiatry. 2022 Nov 19;32(12):2557–67. doi: 10.1007/s00787-022-02110-7 (PMC10682209; doi:10.1007/s00787-022-02110-7)
Supplement: Supplementary file 1 — Supplementary file1 (PDF 206 KB) [file 787_2022_2110_MOESM1_ESM.pdf]

Appendix A. Complete survey (translated)

Q1 Profession[1]
Profession/educational background

Medical doctor/child and adolescent psychiatrist in training
Child and adolescent psychiatrist
Clinical psychologist
Clinical psychologist with specialization in child and adolescent psychology
Nurse
Social worker
Educationalist
Learning disability nurse
Other

Q2 Experience
Total work experience in child and adolescent mental health services

< 5 years
5 - 15 years
> 15 years

Q3 Frequency[1]
How often do you see patients with suspected or diagnosed ADHD?

Approx. daily
Approx. weekly
Approx. monthly
Less [than monthly]

[1] Required question

Q4

Which examinations and screening tools does your team use in the assessment of ADHD?

[Options:** Always – Often – Sometimes – Never**]

Comprehensive review of medical history
Personally meet with patient
Interview/dialogue with caregivers
Interview/dialogue with teacher
Onsite observation of patient in school
Observation of caregiver-child interaction
Observation in playroom if patient is a child
Broad-spectrum assessment tool (e.g., Kiddie-SADS, DAWBA)
Specific symptom scales for ADHD (e.g., ADHD-RS, SNAP-IV)
Trauma assessment
Assessment of patient's drug use
Assessment of caregivers' drug use
Assessment for specific learning disorders
Medical evaluation by physician
Evaluation of sensory disorders/defects
Examination of neuromotoric function with standardized test
Blood tests
Testing response to ADHD medication
Intelligence/cognitive ability test, e.g., WISC
BRIEF
Continuous performance tests, e.g., T.O.V.A.
Other neuropsychological tests
Other (please specify below)

Q5

In your daily work, are there practical limitations that influence the quality of the assessment and differential diagnostic evaluation of ADHD?

[Options:** Always – Often – Sometimes – Never**]

Lack of time
Lack of adequate assessment tools
Insufficient availability of competent professionals
Instruction from leader or local professional practice standards restricts assessment
Other (please specify)

Q6 Certainty

Opinions differ regarding how certain one needs to be in order to diagnose ADHD. What is your opinion?

It must be considered >95 % likely that the patient has ADHD
It must be considered >75 % likely that the patient has ADHD
It must be considered >50 % likely that the patient has ADHD
Diagnosis can be made on an even more uncertain basis if it is assumed that the patient will benefit from pharmacological treatment

Q7 Await

Sometimes the clinical presentation corresponds to ADHD, but ruling out alternative causes for the symptoms is difficult. In the following cases, how often will you postpone making the diagnostic decision?

[Options:** Always – Often – Sometimes – Never**]

Considerable psychosocial challenges
History or presence of trauma
Considerable health problems in close family
Diagnosed sensory deficit (sight, hearing)
Diagnosed neurological conditions
Intellectual functioning deviating from normal range
Other diagnosed developmental disorders
Other (please specify)

Q8 Over/undertreatment

There is disagreement regarding the best way to manage children with ADHD symptoms in the child and adolescent mental health services. Some fear that we are medicalizing normal conditions and that too many receive a diagnosis and medication. Others think the opposite, suggesting more children should be prescribed medication.

What is your opinion about the situation in Norway today?

Overtreatment is most prevalent
Undertreatment is most prevalent
Both over and undertreatment occurs, about equally frequently
Neither over nor undertreatment occurs to a significant degree

Q9 Ideal

Imagine the ideal scenario where all children live under optimal psychosocial conditions, having involved and caring caregivers, receiving appropriate support and accommodations in school, etc. Assume also that health and social services has access to ample resources and competent professionals.

Compared to today, what do you think the prevalence of ADHD among children and adolescents would be in the ideal scenario?

Considerably higher
Somewhat higher
Unchanged
Somewhat lower
Considerably lower
ADHD would not exist

Q10 Medication

We would like your opinions on the following statements regarding the treatment of ADHD.

[Options:]	*Strongly agree*	*Somewhat agree*	*Somewhat disagree*	*Strongly disagree*

Medication is the only real option in the treatment of ADHD
If the patient responds well to medication, there is no need to initiate additional interventions
Psychosocial intervention is an effective form of treatment
I am worried about the long-term consequences of using medication in ADHD treatment
Medication is a prerequisite for enabling psychosocial interventions to work
The side effects of ADHD medications are stronger than many clinicians acknowledge
The use of medications in the treatment of children should be reduced as much as possible
Medication is an effective form of treatment
Medication appears in many instances to become an excuse for institutions and adults surrounding the child not to take further action, so that other important interventions are neglected
If the patient first responds well to psychosocial interventions, initiating medication is unnecessary
Psychosocial interventions are a prerequisite for enabling medication to work

Q11

Lastly, we have two questions regarding the instances where medication is not used in the treatment of ADHD.

In your experience, how often are the following the cause of ***medication not being initiated****?*

[Options:]	*Almost always*	*Often*	*Sometimes*	*Almost never*

The symptoms are managed satisfactorily without medication or other interventions
Clinician does not find sufficient indication for medication
Non-pharmacological treatment interventions are sufficient
Patient disagrees with the diagnosis
Caregiver(s) disagree with the diagnosis
Medication is primarily requested by school staff
Patient worries about side effects
Caregiver(s) worry about side effects
Caregiver(s) worry about medicalization or stigmatization
Patient worries about medicalization or stigmatization
Clinician has reservations toward medication
Patient has reservations toward medication
Caregiver(s) have reservations toward medication
Somatic condition, e.g., the patient has cardiovascular disease
Expectation that patient will not tolerate the medication well
Patient is already prescribed multiple medications
Other (please specify)

Q12
In your experience, how often are the following factors the cause of medication not being continued after initial trial?

[Options:]	*Almost always*	*Often*	*Sometimes*	*Almost never*

Lack of effect
Side effects
Patient's wish
Caregiver's wish
Caregiver has inadequate compliance with treatment
Other (please specify)

Appendix to:

Variation in attitudes toward diagnosis and medication of ADHD: a survey among clinicians in the Norwegian child and adolescent mental health services. *Eur Child Adolesc Psychiatry*

Lyhmann, I; Widding-Havneraas, T; Zachrisson, HD; Bjelland, I; Chaulagain, A; Mykletun, A; Halmøy, A.

Corresponding author: Ingvild Lyhmann, ingvild.lyhmann@helse-bergen.no

Affiliations: Centre for Research and Education in Forensic Psychiatry, Haukeland University Hospital, Bergen, Norway; Department of Clinical Medicine, University of Bergen, Bergen, Norway
