# Supplementary material for: Variation in attitudes toward diagnosis and medication of ADHD: a survey among clinicians in the Norwegian child and adolescent mental health services
Source: Eur Child Adolesc Psychiatry. 2022 Nov 19;32(12):2557–67. doi: 10.1007/s00787-022-02110-7 (PMC10682209; doi:10.1007/s00787-022-02110-7)
Supplement: Supplementary file 2 — Supplementary file2 (PDF 148 KB) [file 787_2022_2110_MOESM2_ESM.pdf]

Appendix B. SEM model output with unstandardized and standardized parameter estimates

SEM model, unstandardized parameters

```
sem (DA -> D1 D2 D3 D4 D5 D6 D7) ///
(MA -> M1 M3 M5 M6 M7 M8 M9), ///
covstruct(_lexogenous, diagonal) method(mlmv) latent(DA MA ) ///
cov( DA@1 DA*MA e.D1*e.D2 e.D1*e.D3 e.D6*e.D7 ///
MA@1 e.M3*e.M5) means( DA@0 MA@0) nocapslatent


Endogenous variables
  Measurement: D1 D2 D3 D4 D5 D6 D7 M1 M3 M5 M6 M7 M8 M9

Exogenous variables
  Latent: DA MA

Fitting saturated model:
Iteration 0:   log likelihood = -9003.3706
Iteration 1:   log likelihood = -8997.7694
Iteration 2:   log likelihood = -8997.6553
Iteration 3:   log likelihood = -8997.6552

Fitting baseline model:
Iteration 0:   log likelihood = -10330.968
Iteration 1:   log likelihood = -10330.943
Iteration 2:   log likelihood = -10330.943

Fitting target model:
Iteration 0:   log likelihood = -9400.7225  (not concave)
Iteration 1:   log likelihood = -9245.5222  (not concave)
Iteration 2:   log likelihood = -9156.9711
Iteration 3:   log likelihood = -9105.7863
Iteration 4:   log likelihood = -9092.2796
Iteration 5:   log likelihood = -9092.0466
Iteration 6:   log likelihood = -9092.0465

Structural equation model                           Number of obs = 674
Estimation method: mlmv

Log likelihood = -9092.0465

 ( 1)  [/]var(DA) = 1
 ( 2)  [/]var(MA) = 1
```

	Coefficient	OIM std. err.	z	P>|z|	[95% conf.	interval]
Measurement						
D1						
DA	.3486842	.024949	13.98	0.000	.299785	.3975834
_cons	.9244126	.0243675	37.94	0.000	.8766532	.972172
D2						
DA	.3888214	.0253654	15.33	0.000	.339106	.4385367
_cons	.8137719	.0252892	32.18	0.000	.764206	.8633377
D3						
DA	.3941736	.0267853	14.72	0.000	.3416754	.4466718
_cons	1.528536	.0265495	57.57	0.000	1.4765	1.580572

D4						
DA	.5187783	.0271534	19.11	0.000	.4655586	.5719981
_cons	1.397599	.0286939	48.71	0.000	1.34136	1.453838
D5						
DA	.5655215	.0250344	22.59	0.000	.5164549	.6145881
_cons	1.198423	.0274681	43.63	0.000	1.144587	1.25226
D6						
DA	.451687	.0262365	17.22	0.000	.4002643	.5031096
_cons	1.116315	.0267977	41.66	0.000	1.063793	1.168838
D7						
DA	.4850119	.0253704	19.12	0.000	.4352869	.5347369
_cons	1.309397	.0266232	49.18	0.000	1.257216	1.361577
M1						
MA	.2450332	.032317	7.58	0.000	.181693	.3083734
_cons	.4735249	.0265114	17.86	0.000	.4215635	.5254862
M3						
MA	.4358736	.0406288	10.73	0.000	.3562427	.5155045
_cons	1.557871	.0336245	46.33	0.000	1.491969	1.623774
M5						
MA	.3174876	.0349667	9.08	0.000	.248954	.3860211
_cons	1.682033	.0282872	59.46	0.000	1.626591	1.737475
M6						
MA	.564611	.04103	13.76	0.000	.4841937	.6450282
_cons	1.320031	.0338208	39.03	0.000	1.253743	1.386318
M7						
MA	.2735698	.0354087	7.73	0.000	.2041701	.3429696
_cons	1.019152	.028904	35.26	0.000	.9625017	1.075803
M8						
MA	.316009	.0367879	8.59	0.000	.2439059	.388112
_cons	.7539231	.0304473	24.76	0.000	.6942474	.8135987
M9						
MA	.2944739	.0392457	7.50	0.000	.2175538	.371394
_cons	.9448263	.0322884	29.26	0.000	.8815422	1.00811
var(e.D1)	.2772418	.0159373			.2477007	.3103059
var(e.D2)	.2780465	.0173755			.2459942	.3142752
var(e.D3)	.3153143	.0194907			.2793366	.3559258
var(e.D4)	.2727252	.0189302			.2380359	.31247
var(e.D5)	.1825767	.0157442			.1541854	.2161959
var(e.D6)	.2771729	.0181754			.2437439	.3151866
var(e.D7)	.2384281	.0165656			.2080738	.2732106
var(e.M1)	.4103434	.0244338			.3651429	.4611392
var(e.M3)	.5654357	.0382852			.495164	.64568
var(e.M5)	.42057	.0273808			.3701873	.4778097
var(e.M6)	.4454257	.0397492			.3739514	.5305612
var(e.M7)	.4812024	.0289572			.4276665	.5414399
var(e.M8)	.5214561	.0318201			.462675	.587705
var(e.M9)	.6110143	.0362391			.5439599	.6863346
var(DA)	1	(constrained)				
var(MA)	1	(constrained)				
cov(e.D1,e.D2)	.1435695	.0134754	10.65	0.000	.1171583	.1699808
cov(e.D1,e.D3)	.0560801	.0110049	5.10	0.000	.0345109	.0776494
cov(e.D6,e.D7)	.1053647	.0138636	7.60	0.000	.0781926	.1325367
cov(e.M3,e.M5)	.1694005	.0252511	6.71	0.000	.1199093	.2188918
cov(DA,MA).	.4027982	.0478745	8.41	0.000	.3089659	.4966305

LR test of model vs. saturated: chi2(72) = 188.78 Prob > chi2 = 0.0000

SEM model, standardized parameters

```
sem (DA -> D1 D2 D3 D4 D5 D6 D7) ///
(MA -> M1 M3 M5 M6 M7 M8 M9), ///
covstruct(_lexogenous, diagonal) method(mlmv) latent(DA MA ) ///
cov( DA@1 DA*MA e.D1*e.D2 e.D1*e.D3 e.D6*e.D7 ///
MA@1 e.M3*e.M5) means( DA@0 MA@0) nocapslatent ///
standardized


Endogenous variables
  Measurement: D1 D2 D3 D4 D5 D6 D7 M1 M3 M5 M6 M7 M8 M9

Exogenous variables
  Latent: DA MA

Fitting saturated model:
Iteration 0:   log likelihood = -9003.3706
Iteration 1:   log likelihood = -8997.7694
Iteration 2:   log likelihood = -8997.6553
Iteration 3:   log likelihood = -8997.6552

Fitting baseline model:
Iteration 0:   log likelihood = -10330.968
Iteration 1:   log likelihood = -10330.943
Iteration 2:   log likelihood = -10330.943

Fitting target model:
Iteration 0:   log likelihood = -9400.7225  (not concave)
Iteration 1:   log likelihood = -9245.5222  (not concave)
Iteration 2:   log likelihood = -9156.9711
Iteration 3:   log likelihood = -9105.7863
Iteration 4:   log likelihood = -9092.2796
Iteration 5:   log likelihood = -9092.0466
Iteration 6:   log likelihood = -9092.0465

Structural equation model                          Number of obs = 674
Estimation method: mlmv

Log likelihood = -9092.0465

 ( 1)  [/]var(DA) = 1
 ( 2)  [/]var(MA) = 1
```

Standardized	Coefficient	OIM std. err.	z	P>|z|	[95% conf.	interval]
Measurement						
D1						
DA	.5521314	.0312781	17.65	0.000	.4908275	.6134354
_cons	1.463781	.0552059	26.51	0.000	1.355579	1.571983
D2						
DA	.5934796	.0299261	19.83	0.000	.5348255	.6521338
_cons	1.242105	.0513869	24.17	0.000	1.141389	1.342822
D3						
DA	.5745415	.0307952	18.66	0.000	.514184	.634899
_cons	2.227971	.0722038	30.86	0.000	2.086454	2.369488
D4						
DA	.7047577	.0246295	28.61	0.000	.6564848	.7530306
_cons	1.898631	.065287	29.08	0.000	1.770671	2.026592
D5						
DA	.7978625	.0206478	38.64	0.000	.7573937	.8383314
_cons	1.690788	.060418	27.98	0.000	1.572371	1.809205

D6						
DA	.6511446	.0274026	23.76	0.000	.5974366	.7048526
_cons	1.609262	.0584756	27.52	0.000	1.494652	1.723872
D7						
DA	.7047205	.024738	28.49	0.000	.6562349	.7532061
_cons	1.902549	.0649566	29.29	0.000	1.775236	2.029861
M1						
MA	.3572715	.0442704	8.07	0.000	.270503	.4440399
_cons	.6904246	.0430247	16.05	0.000	.6060977	.7747515
M3						
MA	.5014942	.0414454	12.10	0.000	.4202626	.5827257
_cons	1.792408	.0625061	28.68	0.000	1.669899	1.914918
M5						
MA	.4396979	.0442702	9.93	0.000	.3529299	.5264659
_cons	2.329497	.0757595	30.75	0.000	2.181011	2.477983
M6						
MA	.6458662	.0390259	16.55	0.000	.5693768	.7223556
_cons	1.510001	.0566647	26.65	0.000	1.398941	1.621062
M7						
MA	.3668716	.0444832	8.25	0.000	.2796861	.454057
_cons	1.366737	.0538633	25.37	0.000	1.261167	1.472307
M8						
MA	.400906	.0431015	9.30	0.000	.3164285	.4853835
_cons	.9564673	.0466249	20.51	0.000	.8650841	1.047851
M9						
MA	.3525359	.0441984	7.98	0.000	.2659086	.4391632
_cons	1.13112	.0494899	22.86	0.000	1.034121	1.228118
var(e.D1)	.6951509	.0345393			.6306469	.7662524
var(e.D2)	.6477819	.0355211			.5817726	.7212809
var(e.D3)	.6699021	.0353863			.6040158	.7429753
var(e.D4)	.5033166	.0347156			.4396739	.5761716
var(e.D5)	.3634154	.0329481			.3042505	.4340856
var(e.D6)	.5760107	.0356861			.510147	.6503778
var(e.D7)	.503369	.0348667			.4394673	.5765626
var(e.M1)	.8723571	.0316331			.8125092	.9366132
var(e.M3)	.7485036	.0415693			.6713069	.8345775
var(e.M5)	.8066657	.038931			.73386	.8866945
var(e.M6)	.5828568	.050411			.4919736	.690529
var(e.M7)	.8654052	.0326392			.8037408	.9318007
var(e.M8)	.8392744	.0345593			.7742006	.9098178
var(e.M9)	.8757184	.0311631			.8167213	.9389773
var(DA)	1	(constrained)				
var(MA)	1	(constrained)				
cov(e.D1,e.D2)	.5170997	.0304622	16.98	0.000	.457395	.5768044
cov(e.D1,e.D3)	.1896741	.0349009	5.43	0.000	.1212696	.2580785
cov(e.D6,e.D7)	.4098651	.0380125	10.78	0.000	.3353619	.4843683
cov(e.M3,e.M5)	.3473794	.0401006	8.66	0.000	.2687838	.425975
cov(DA,MA).	.4027982	.0478745	8.41	0.000	.3089659	.4966305

LR test of model vs. saturated: chi2(72) = 188.78 Prob > chi2 = 0.0000

Appendix to:

Variation in attitudes toward diagnosis and medication of ADHD: a survey among clinicians in the Norwegian child and adolescent mental health services. *Eur Child Adolesc Psychiatry*

Lyhmann, I; Widding-Havneraas, T; Zachrisson, HD; Bjelland, I; Chaulagain, A; Mykletun, A; Halmøy, A.

Corresponding author: Ingvild Lyhmann, ingvild.lyhmann@helse-bergen.no

Affiliations: Centre for Research and Education in Forensic Psychiatry, Haukeland University Hospital, Bergen, Norway; Department of Clinical Medicine, University of Bergen, Bergen, Norway
